# Clinical Relation Extraction Toward Drug Safety Surveillance Using Electronic Health Record Narratives: Classical Learning Versus Deep Learning

**DOI:** 10.2196/publichealth.9361

**Published:** 2018-04-25

**Authors:** Tsendsuren Munkhdalai, Feifan Liu, Hong Yu

**Affiliations:** ^1^ Department of Quantitative Health Sciences University of Massachusetts Medical School Worcester, MA United States; ^2^ Department of Computer Science University of Massachusetts Lowell Lowell, MA United States; ^3^ The Bedford Veterans Affairs Medical Center Bedford, MA United States

**Keywords:** medical informatics applications, drug-related side effects and adverse reactions, neural networks, natural language processing, electronic health records

## Abstract

**Background:**

Medication and adverse drug event (ADE) information extracted from electronic health record (EHR) notes can be a rich resource for drug safety surveillance. Existing observational studies have mainly relied on structured EHR data to obtain ADE information; however, ADEs are often buried in the EHR narratives and not recorded in structured data.

**Objective:**

To unlock ADE-related information from EHR narratives, there is a need to extract relevant entities and identify relations among them. In this study, we focus on relation identification. This study aimed to evaluate natural language processing and machine learning approaches using the expert-annotated medical entities and relations in the context of drug safety surveillance, and investigate how different learning approaches perform under different configurations.

**Methods:**

We have manually annotated 791 EHR notes with 9 named entities (eg, medication, indication, severity, and ADEs) and 7 different types of relations (eg, medication-dosage, medication-ADE, and severity-ADE). Then, we explored 3 supervised machine learning systems for relation identification: (1) a support vector machines (SVM) system, (2) an end-to-end deep neural network system, and (3) a supervised descriptive rule induction baseline system. For the neural network system, we exploited the state-of-the-art recurrent neural network (RNN) and attention models. We report the performance by macro-averaged precision, recall, and F1-score across the relation types.

**Results:**

Our results show that the SVM model achieved the best average F1-score of 89.1% on test data, outperforming the long short-term memory (LSTM) model with attention (F1-score of 65.72%) as well as the rule induction baseline system (F1-score of 7.47%) by a large margin. The bidirectional LSTM model with attention achieved the best performance among different RNN models. With the inclusion of additional features in the LSTM model, its performance can be boosted to an average F1-score of 77.35%.

**Conclusions:**

It shows that classical learning models (SVM) remains advantageous over deep learning models (RNN variants) for clinical relation identification, especially for long-distance intersentential relations. However, RNNs demonstrate a great potential of significant improvement if more training data become available. Our work is an important step toward mining EHRs to improve the efficacy of drug safety surveillance. Most importantly, the annotated data used in this study will be made publicly available, which will further promote drug safety research in the community.

## Introduction

### Background and Significance

Prescription drug safety represents a major public health concern [1]. An adverse drug event (ADE) is “an injury resulting from medical intervention related to a drug” [2]. ADEs are common and occur in approximately 2-5% of hospitalized adult patients [2-5]. Each ADE is estimated to increase the length of a hospital stay by more than 2 days and hospital cost by more than US $3200 [4,6]. Severe ADEs rank among the top 4 or 6 leading causes of death in the United States [7]. Prevention, early detection, and mitigation of ADEs could save both lives and resources [6,8,9].

Due to the limited number of participants and inclusion or exclusion criteria reflecting specific subject characteristics, premarketing randomized clinical trials frequently miss ADEs [1], and thus, postmarketing drug safety surveillance [10] is vitally important for health care and patient safety. The Food and Drug Administration (FDA) maintains an adverse event reporting system called the Food and Drug Administration Adverse Event Reporting System for postmarketing safety surveillance, but it faces challenges including underreporting [11,12] and missing important patterns of drug exposure [13]. Other resources have been shown to be useful for identifying ADEs, including biomedical literature [14] and social media [15-18]. However, biomedical literature has been shown to identify mostly a limited set of rare ADEs [19]. Social media has its own challenges, such as missing important drug exposure patterns and generalizing system to deal with data heterogeneity [17].

It is well known that electronic health records (EHRs) contain rich ADE information and are an important resource for drug safety surveillance [2,20,21]. Since 2009, the FDA has invested in facilitating the use of routinely collected EHR data to perform active surveillance of the safety of marketed medical products [22]. Existing ADE-targeted observational studies have focused on structured EHR data for obtaining ADE information [23-25]; however, ADEs are often buried in the EHR narratives and not recorded in structured data. Manual abstraction of data from EHR notes [5,26] remains a costly and significant impediment to drug safety surveillance research. Exploring natural language processing (NLP) approaches for efficient, accurate, and automated ADE detection can provide significant cost and logistical advantages over manual chart review or voluntary reporting.

### Mining Clinical Narratives for ADE Detection

Quite a few NLP approaches have been explored for mining ADE information from unstructured data of the aforementioned sources, such as biomedical literature [27,28], social media [29], FDA event reporting system narratives [30], and EHRs [31-40]. The 2009 i2b2 (Informatics for Integrating Biology and the Bedside) medication challenge [41] and the 2010 i2b2 relation challenge [42] plays an important role to promote methodology advancement in this field. Existing studies are limited to detect only on the document level by identifying discharge summaries that contains ADE [31], or mainly focus on detecting entities representing relevant events (eg, adverse events and medication events) [32,33,43], or deal with only intrasentential relations [42], or identify relations purely based on statistical association analysis among drug and outcome concepts, which are recognized by mapping free clinical text onto medical terminology [37-40]. Henriksson et al [35] explored traditional random-forest algorithm to identify relations between drugs and disorders (or findings) on Swedish clinical notes, and reported that the intersentential relations are challenging and hard to detect.

Recently, deep learning with neural networks has received increasing attention in NLP tasks [44,45], and for relation extraction, the state-of-the-art systems are based on 2 networks: recurrent neural networks (RNNs) [46,47] and convolutional neural networks (CNNs) [48], and an end-to-end relation extraction model [49] obtained competitive performance on several datasets. So far, there is less related work on evaluating deep learning methods on ADE relation extraction. Li et al [50] proposed a bidirectional LSTM to extract ADE relations from biomedical literature. As the model is dependent on the parsing of a sentence, it is difficult to apply that on clinical notes which contain more abbreviations and ungrammatical language expressions. In clinical domain, Lv et al [51] combined autoencoder with conditional random fields, and Sahu et al [52] proposed a domain invariant CNNs for ADE extraction on the i2b2 data. All the 3 studies are limited to extract relations within 1 sentence.

### Objective

In this study, we investigate ADE-relevant relation extraction on both intra- and intersentential settings. To this end, we have built a benchmark corpus consisting of clinical notes where medical concepts related to ADE and their relations were annotated via a manual chart review. Then, we experimented with 3 supervised machine learning approaches for ADE relation identification from clinical notes. The first approach is based on rule induction, which is similar to supervised descriptive rule induction [53] but is relatively simple. Rules for each relation type are automatically induced based on the corresponding descriptive statistics obtained from the training data, and then those rules are used to classify new entity pairs. Our second approach uses a classical support vector machines (SVM)-based machine learning model. Our third approach is based on deep learning neural networks, which explore RNNs with attention mechanisms. In addition to benchmark the overall performance, we empirically analyzed how well deep learning models are in terms of recognizing long-distance relations, and how the training data size affects learning performance on clinical data. Compared with previous studies, the main contributions of this work are as follows:

- We build a new annotated benchmark corpus of EHR notes for ADE information extraction. Compared with the existing i2b2 data, this corpus contains much richer annotations related to ADE research, for example, all the medications are profiled with attributes enabling ADE connected to a specific dose of medication (note that many ADEs are caused by high dosage); severity concepts are also annotated and associated with ADEs.
- The annotated data in this study will be shared with the community to further promote research for drug safety surveillance.
- It is the first attempt to investigate and evaluate modeling 7 heterogeneous clinical relations in a single framework: relations between medication and its attributes, relations between ADE and its severity, relations between medication and ADE, and relations between medication and indication.
- We explored RNNs and attention mechanisms for clinical relation extraction beyond sentence boundaries, and investigate how the length between two entities affects the performance for different learning models. To our knowledge, this is the first study of applying deep learning approaches on both inter- and intrasentential relation extraction using EHR data.

## Methods

### Data Annotation

The annotated corpus contains 791 English EHR notes from cancer patients, which were randomly sampled from people who have been diagnosed with hematological malignancy and have drug exposure to one or more of the 12 cancer drugs of interest, including Romidepsin, Rituximab, Brentuximab vedotin, Ponatib, Carfilzomib. All the notes are longitudinal and no note type filtering was performed. We manually annotated 8 named entities and 7 relation types among them: *Dosage-Medication, Route-Medication, Frequency-Medication, Duration-Medication, Medication-Indication, Medication-ADE*, and *Severity-ADE*. One named entity that is not involved in relations is “other signs and symptoms.” Our annotation guidelines are an extension of the i2b2 annotation guidelines [42] and have been iteratively developed by domain experts. Unlike other clinical corpora that annotate entity relations at the sentence level, we annotated entity relations beyond sentence boundaries. Each EHR note was annotated by at least 2 annotators, and the interannotator agreement of .93 kappa was achieved on our annotations.

The resulting annotated data consisted of 667,061 tokens, 48,803 entity mentions (61.7 per note), and 16,022 entity relations (20.3 per note). The relation distributions in these datasets are reported in the last column of Table 1. *Frequency*, *dosage*, and *indication* are the most frequent relations, whereas *duration* and *adverse* relations are less frequent in the corpus. We split the corpus into 602/95/94 train/develop/test sets.

**Figure 1:**
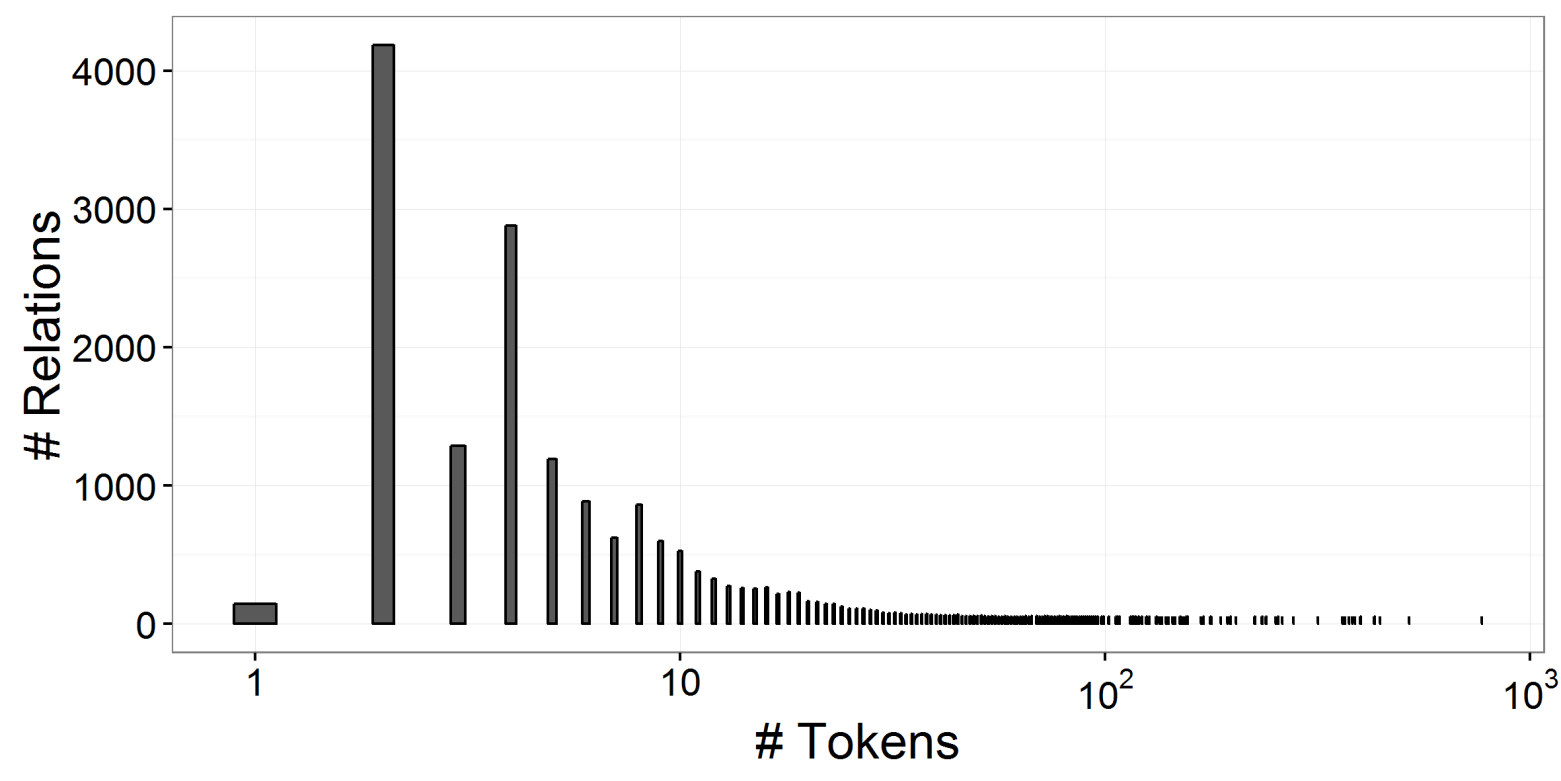
The distribution of relation token distance.

Figure 1 shows the distribution of relation token distance (the number of tokens between a relation entity mention pair). As shown in Figure 1, most relations occurred within a window of up to 9 tokens. On the other hand, some relations connected entities across multiple sentences. The average relation token distance was 7, and the maximum distance was 769.

**Figure 2:**
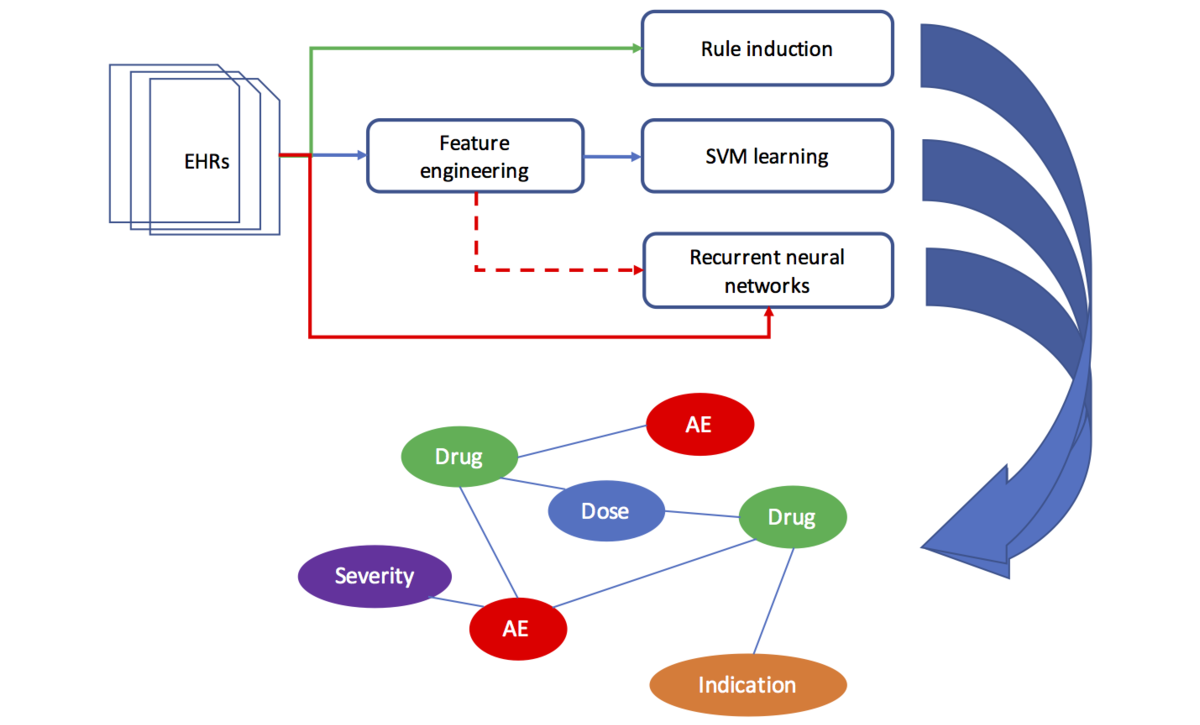
Experimental workflow for adverse drug event (ADE) detection. EHRs: electronic health records; SVM: support vector machines; AE: adverse events.

To formulate the relation identification task, our goal was to learn a function *f* (*x*) that mapped an input entity pair (*e*_l_, *e*_r_) to a relation type *y* ∈ *Y*, where *Y* is the set of all possible relation types including *None*, which in our system denotes the existence of no relation between an entity pair. An entity *e*_i_ ∈ *E* is any observed entity mention within a document *d* ∈ *D*. The input entity pair (*e*_l_, *e*_r_) is sampled from all possible entity pairs *E* x *E* within the document and is labeled with a relation type if a true relation holds for it; otherwise, it is labeled *None*. The mention pair and the document within which that pair occurs form a machine learning example *x* in our task. We implemented and evaluated 3 supervised machine learning approaches as described below, and the experiment workflow is shown in Figure 2.

**Table 1:** Clinical relation types in our corpus. Entity mentions forming relations are in italics.

Relation	Description	Example	#relations^a^
*Dosage*	An attribute of a medication: the amount of the medication to be taken	She receives *Albuterol 2 puffs* p.o. q4-6h	2643/336/409
*Route*	An attribute of a medication: how the medication is administered	She receives *Albuterol* 2 puffs *p.o.* q4-6h	1908/269/332
*Frequency*	An attribute of a medication: frequency of the administration	She receives *Albuterol* 2 puffs p.o. *q4-6h*	2691/351/451
*Duration*	An attribute of a medication	The patient was treated with *ampicillin* for *2 weeks*	493/95/110
*Indication*	A causal relation between a medication and indication: why the drug is taken	He later received *chemotherapy* for his *lung cancer*	2301/264/379
*Adverse Event*	A causal relation between a medication and an injury: the consequence of a medication	Patient’s death was due to *anaphylactic shock* caused by the intravenously administered *penicillin*	717/134/134
*Severity*	The attribute of an adverse event	He has *severe diarrhea*	1505/259/241

^a^the number of relations for each type (train/develop/test).

### Induction Rule Baseline

Our first supervised approach used automatically induced rules from the training data, motivated by the observation that the distance between 2 entities was a potentially strong indicator of their relations. For example, we observed that drug attributes typically followed drug names and, in contrast, the distance between adverse drug events and their drugs was relatively far. Therefore, our rule-induction classifier was based on the token distance between 2 entities.

Formally, the classifier considered an entity pair (*e*_l_, *e*_r_) that occurred within a certain distance as a true relation, and the pair was assigned one of the positive relation types, . For training, we calculated the average token distance of the entity pairs for each relation type. We then defined 7 different token distance bins by using these average distances and assigning a single positive relation label to each bin. During prediction, we chose one of the relation labels if the token distance of 2 entities fell in the corresponding bin. For example, if the average token distance for *Severity* relations was 3 and for *Frequency* was 7, we then had 2 bins, { *n* | 0 < *n* ≤ 3} and { *n* | 3 < *n* ≤ 7} (*n* was the token distance). If the token distance *n* between an entity pair was in the first bin, the entity pair was given the label *Severity*; otherwise, it was labeled *Frequency* or *None*. We considered an entity pair as *None* relation if their token distance did not belong to any one of the predefined bins.

### Support Vector Machines System

We identified a set of rich learning features to build a linear kernel SVM classifier. We chose linear SVM due to its ability to accommodate a large feature space. The features we explored are described below.

*Document-level features* consisted of the frequencies of a specific entity and entity type in a document.

*Relation-specific features* were specific to an entity pair being considered for classification. The features were as follows:

- token distance between the 2 entities
- number of clinical entities between the 2 entities
- *n-grams* (*1, 2, 3-grams*) between the 2 entities
- *n-grams* (*1, 2, 3-grams*) of surrounding tokens of the 2 entities. The surrounding tokens were within a window size, which was defined empirically in our experiment.

*Entity-level features* defined how likely an individual entity mention was involved in a relation:

- one-hot encoding of the left entity type, *e*_l_
- one-hot encoding of the right entity type, *e*_r_
- character *n-grams* (*2, 3-grams*) of the named entities.

*Semantic features* were derived using the MetaMap tool from National Library of Medicine. Specifically, we mapped entity mentions and their surrounding context to their UMLS(Unified Medical Language System) concepts, preferred terms, and semantic types. We renormalized the concept IDs (identifiers) to their corresponding semantic type names and included both shortened and multiword forms of the semantic types in the feature set. We set the window size of the surrounding context to 10 in the MetaMap tool.

*Word representation features* were generated to overcome the data sparsity challenge. We explored word clustering and word vector representation features that have been shown to improve performance for chemical and biomedical named-entity recognition tasks [54,55]. In particular, we used the Brown clustering model and Word Vector Classes as word clustering features and applied raw word embedding as word vector features.

We trained the Brown cluster model [56] on a large collection of biomedical text. We then obtained the cluster label prefixes (ie, the top levels of the cluster hierarchy) with 4, 6, 10, and 20 lengths from the Brown model as features for the context of each entity mention. We empirically set the context window size to 10 in this study. To learn broader contextual information, we also explored recently introduced skip-gram model [57]. The skip-gram model is used to predict the contextual words given an input token, and this yielded a dense word embedding for the token that effectively carried its syntactic and semantic information. We first built a skip-gram model on a large unlabeled text consisting of the PubMed abstracts and the EHRs [43], and an additional set of ~2 million PubMed Central full articles. The word embedding induced by the skip-gram model were then clustered into 300 different groups by using a K-means algorithm to obtain cluster labels that we called Word Vector Classes (WVCs). As with the Brown model features, we mapped the entity mention context to their WVCs and included these WVCs in the feature set. We also used the raw word embedding as word representation features in our model, which provided a fine-grained latent feature of word semantic and syntactic information.

The character and word *n-grams* were converted into *TF-IDF(term frequency-inverse document frequency)* weights based on the training set. We stored the *TF-IDF* weights and used them to extract features from the development and test sets. We did not involve the development and test sets in the *n-gram* extraction and the *TF-IDF* calculation to ensure that our models and the features were not biased. We did not extract any sentence-specific features, which allowed us to classify intra- and intersentential relations jointly with a single SVM model.

### End-to-End Deep Neural Networks

We explored LSTM and attention-based neural network methods to classify clinical relations in an end-to-end fashion [58] without feature engineering. The reason behind this choice is based on reported advantages of RNNs over CNNs in relation extraction tasks [59,60].

LSTM is a variation of RNN models and was introduced to solve the gradient vanishing problem [61,62]. It can model long-term dependencies with its internal memory, and it achieved notable success with NLP tasks including machine translation [63], speech recognition [64], and textual entailment recognition [65]. The LSTM can effectively learn vector representations for various levels of linguistic units to facilitate different classification tasks. The attention mechanism can help LSTM construct a better representation by selecting important context in an EHR document. As it is computationally expensive to use the whole document for learning the representations, we focused on text windows associated with the 2 entities in our model.

Let *x*_t_, *h*_t_, and *c*_t_ be the input, output, and cell state, respectively, at time step *t*. Given a window of token representations (ie, word embeddings) *x*_1_,…, *x*_l_ (*x*_l_ is the head token for the entity *e*_l_ and *L* is the window size), an LSTM with hidden size *k* computes a sequence of the outputs *h*_1_,…, *h*_l_ and another sequence of the cell states *c*_1_,…, *c*_l_ as: σ

*i*_t_= σ (*W*_1_^lstm^*x*_t_+ *W*_2_^lstm^*h*_t-1_+ *b*_1_^lstm^) (1)

*i*_t_^'^= *tanh* (*W*_3_^lstm^*x*_t_+ *W*_4_^lstm^*h*_t-1_+ *b*_2_^lstm^) (2)

*f*_t_= σ (*W*_5_^lstm^*x*_t_+ *W*_6_^lstm^*h*_t-1_+ *b*_3_^lstm^) (3)

*o*_t_= σ (*W*_7_^lstm^*x*_t_+ *W*_8_^lstm^*h*_t-1_+ *b*_4_^lstm^) (4)

*c*_t_ = *f*_t_ ⊙ *c*_t-1_ + *i*_t_ ⊙ *i*_t_^’^ (5)

*h*_t_ = *o*_t_ ⊙ *tanh* (*c*_t_) (6)

where *W*_1_^lstm^,…, *W*_8_^lstm^ ∈ *R*^k×k^ and *b*_1_^lstm^,…, *b*_4_^lstm^ ∈ *R*^k^ are the training parameters, and σ and ⊙ denote the element-wise sigmoid function and the element-wise vector multiplication, respectively.

As described by the equations, the memory cell *c*_t_ and hidden state *h*_t_ were updated by reading a word token *x*_t_ at a time. The memory cell *c*_t_ then learns to remember the contextual information that is relevant to the entity mention. This information is then provided to the hidden state *h*_t_ by using a gating mechanism, and the last hidden state *h*_l_ summarizes all the relevant information for the sequence. *i*_t_, *f*_t_, and *o*_t_ are called gates whose values are defined by the nonlinear combination of the previous hidden state *h*_t-1_ and the current input token *x*_t_ and range from 0 to 1. The input gate *i*_t_ controls how much information needs to flow into the memory cell, whereas the forget gate *f*_t_ decides what information needs to be erased from the memory cell. The output *o*_t_ finally produces the hidden state for the current input token.

We further used the output *h*_l_ and *h*_r_ corresponding to the input token heads of the entity pair *e*_l_ and *e*_r_ as the entity representations. The representation *h*_r_ for entity *e*_r_ was obtained similarly by reading its token window with another LSTM. The representations *h*_l_ and *h*_r_ were then composed by using a function *g* (*h*_l_, *h*_r_) to produce a relation representation *r*_lr_. We used a multilayered perceptron (MLP) with a concatenated input for *g* (*h*_l_, *h*_r_) in our model, defined as:

*r*_lr_= *g* (*h*_l_, *h*_r_) (7)

*g* (*h*_l_, *h*_r_) = *tanh* (*W*_mlp_[*h*_l_; *h*_r_] + *b*_mlp_) (8)

where [*h*_l_; *h*_r_] is the concatenation operation, *W*^mlp^ ∈ *R*^k×|^^Y^^|^ is the projection matrix, and *b*^mlp^ ∈ *R*^|Y^^|^ is the bias vector trained from the data. Finally, the relation representation *r*_lr_ was input to the *softmax* layer to normalize the probability distribution over possible relation types *Y*. The whole network was trained by a backpropagation algorithm by minimizing the cross-entropy loss between the predicted probabilities and the correct labels.

We also experimented LSTM with the attention mechanism, which is expected to solve the issue of the information bottleneck in RNNs [66]. When RNNs process long text, they encounter a practical difficulty; they must compress the text into a single vector with a fixed size. The purpose of the attention mechanism is to exploit the task-relevant outputs in the past time scales and the current output vector to dynamically refine the final vector representation so that the constructed presentation becomes more informative.

We used a standard global attention, which has shown to be state-of-the-art in a variety of NLP tasks: machine translation [66], question answering [67], textual entailment [68], and constituency parsing [69]. In addition to the last output vectors *h*_l_ and *h*_r_, the global attention explicitly considered all the previous output vectors *h*_1_,…, *h*_l-1_ and *h*_1_,…, *h*_r-1_ to construct attention-weighted representations of the entities *e*_l_ and *e*_r_.

Concretely, let *S* ∈ *R*^k×l^ be a matrix of the output vectors *h*_1_,…, *h*_l_ and *o*_l_ ∈ *R*^l^ be a vector of ones. An attention weight vector *a*, an attention representation *z*, and the final entity representation *h*_l_^’^ were defined as:

*M* = *tanh* (*W*_1_^at^*S* + *W*_2_^at^*h*_l_⊕ *o*_l_) (9)

*a* = *softmax* (*w*^T^*M*) (10)

z= *Sa*^T^ (*11*)

*h*_l_^’^= *tanh* (*W*_3_^at^*z* + *W*_4_^at^*h*_l_) (12)

where *W*_1_^at^, *W*_2_^at^, *W*_3_^at^, *W*_4_^at^ ∈ *R*^k×k^ are learnable matrices and *w*^T^ is the transpose of the learnable vector *w* ∈ *R*^k^. With the outer product *W*_2_^at^*h*_l_⊕ *o*_l_, we repeated the transformed vector of *h*_l_*l* times and then combined the resulting matrix with the projected output vectors. The entity representation *h*_r_^’^ for entity *e*_r_ was obtained similarly. As for the LSTM-based relation representation, the compositions of the representations were input to an MLP for relation classification.

We also used the bidirectional version of the aforementioned models by feeding concatenated outputs of the forward and backward LSTM. Due to the concatenated outputs, the size of the *W* matrices and *w* vector now become 2 *k* × 2 *k* and 2 *k*, respectively, increasing the number of parameters to be trained. We have previously shown that bidirectional LSTM outperformed the LSTM models for medication and adverse drug event named-entity recognition tasks in EHRs [43].

### Experimental Setup and Evaluation Metrics

As noted previously, we split the corpus into 602/95/94 train/development/test sets. To cast the task as a multiclass classification problem, we generated *None* relations (negative examples) by replacing one of the entity mentions of a true relation with another entity. In doing so, the only constraint was that the new relation should not exist in the true relation corpus set and the rest should be learned from the data. This process gave us additional negative relation instances of 1,190,328/144,338/202,065 for the train/development/test sets, respectively. For this SVM model, we carried out a grid search over its hyperparameters by using the development set for evaluation. Once the best parameters were found, the final SVM model was learned using the optimized hyperparameters on both the training and development sets.

We used ADAM (adaptive moment estimation) [70] for optimization of the neural models. The size of the LSTM hidden units was set to 100. An additional layer was used to map word vectors to the LSTM input. We used a pretrained word2vec model with a size of 300 [43] for word embedding. All neural models were regularized by using 20% input and 30% output dropouts [71] and an *l*_2_ regularizer with strength value 1e-3. The neural models were trained only on the training set. We used the development set to evaluate them for each epoch to choose the best model. The unidirectional models were given 30 epochs and the attentional and bidirectional models were given 60 epochs to converge to an optimum. The final performance of the methods was reported and compared by using the test set.

Our experiment was guided by macro-averaged precision, recall, and *F* 1-score in terms of positive relation types. False negative (*FN*) and false positives (*FP*) are incorrect negative and positive predictions, respectively. True positive (*TP*) results correspond to correct positive predictions, which were actually correct predictions. Recall (*r*) denotes the percentage of correctly labeled positive results over all positive cases and is calculated as: *r=TP/(TP+FN).* Precision (*p*) is the percentage of correctly labeled positive results over all positive-labeled examples and is calculated as: *p=TP/(TP+FP).* The *F* 1-measure is the harmonic average of precision and recall, and a balanced *F* 1-score is expressed as: *F*_1_*=2pr/(p+r).*

## Results

This section presents the results of implementing our relation identification systems. We analyzed the performance of each model and the effects of their free parameters.

### The Rule Induction Baseline

For this baseline, the distance bins were defined by using the training data. If the token distance of an entity pair did not belong to any of the bins, it was labeled as a *None* relation. This baseline achieved an 7.47% overall F1-score on the test set. Detailed results are shown in Table 2. The performance was low, as the method was very simple. The *Dosage* relation type achieved the highest F1-score (30%) among different relations.

### Support Vector Machines–Based Pipeline System

We performed down-sampling for the negative relations (*None* relations) with varying keep rates to study how the performance changed for different distributions of *None* examples involved in the training set. The development and test sets were kept the same.

Table 3 reports the overall F1-score of our SVM model. A higher keep rate means that we used more negative relations in the training set, and that the higher keep rate yielded a better result on the test set in our experiment. We obtained the highest performance with the keep rate value equal to 80% in our SVM model. The training set for this run consisted of 1,096,600 instances, of which 964,520 were *None* relations. In Table 4, we show the detailed performance metrics for this model for each relation type when evaluated on the test set. The F1-scores for most relation types were over 80% with *Route* relation achieving the best of 96%, and the recall of our clinical relation extractor was relatively high. However, the performance of the *Indication* and *Adverse* relations were not as high as those of the other relations, and *Indication* showed the worst score of 75%. We observed that 2 entities forming these types of relations tended to be far away from each other and spanned multiple sentences (the average token distance was 19 and 14, and the maximum was 518 and 769). The long distance makes this relation more difficult to detect than other relations.

**Table 2:** Results (%) of rule induction classifier on test set.

Relation	Precision	Recall	F1-score
None	100	94	97
Dosage	20	63	30
Route	7	31	11
Frequency	2	7	3
Duration	1	4	1
Indication	1	14	2
Adverse	1	24	1
Severity	0	0	0
Overall	4.57	20.42	7.47

**Table 3:** Overall F1-scores (%) of support vector machines system. Keep rate for negative down-sampling is varied.

Keep rate	Train	Development	Test
0.1	99.99	99.97	82.46
0.3	99.96	99.93	87.84
0.5	99.94	99.86	89.0
0.8	99.89	99.8	*89.1* ^a^

^a^Best score on test data are highlighted in italics.

### End-to-End Deep Neural Networks

We also examined the performance of the neural network models. Notably, by leveraging recent advances in deep learning, including efficient representation learning and attention mechanisms, we addressed the problem without any hand-engineered features.

As stated earlier in the Methods section, we used a free parameter window size to determine how much local context is considered for entity representation in neural network models. We first examined the effect of this parameter by training the unidirectional LSTM-based model that was the least complex and the fastest to train and to test. The keep rate for down-sampling was set to 0.1 and the window sizes 5, 10, 30, 50, and 70 were studied. Table 5 presents the results.

When we considered more context with a larger token window, the performance of the LSTM-based relation extractor improved. However, there appeared to be a small drop starting at the point where size is equal to 50, suggesting that large window size may introduce contextual noise into the model. In addition, the training and test time dramatically increased with the large windows; therefore, we set the window size to 30 in our experiments, unless specified.

We conducted a similar group of experiments to observe how the different down-sampling rates affected the model learning. Again, we used an LSTM-based model to report the results, because it was the least complex and fastest to train. The results are presented in Table 6. This time we observed a different pattern of results. The training error kept decreasing as we included more negative examples in the training set. However, with the keep rate of 0.8, it started showing decreasing performance on the development and the test sets. We used a down-sampling keep rate of 0.5 throughout the experiment.

**Table 4:** Results (%) of the best performing support vector machines model on test set. Keep rate=0.8.

Relation	Precision	Recall	F1-score
None	100	100	100
Dosage	85	91	88
Route	96	97	96
Frequency	93	97	95
Duration	89	93	91
Indication	72	77	75
Adverse	85	84	85
Severity	95	94	95
Overall	87.85	90.42	89.1

**Table 5:** Overall F1-score of the long short-term memory (LSTM)–based model. Keep rate=0.1.

Window size	Train	Development	Test
5	24.05	14.09	14.58
10	23.92	14.85	14.56
30	37.40	21.77	*22.59* ^a^
50	32.1	17.15	18.43
70	27.62	15.04	15.93

^a^Best score on test data are highlighted in italics.

**Table 6:** Overall F1-score of the long short-term memory (LSTM)–based model. Keep rate for negative down-sampling is varied. Window size=10.

Keep rate	Train	Development	Test
0.1	23.92	14.85	14.56
0.3	38.91	35.18	37.21
0.5	51.25	39.02	*39.45* ^a^
0.8	24.82	23.65	21.11

^a^Best score on test data are highlighted in italics.

**Table 7:** Overall F1-score (%) of long short-term memory (LSTM) and attention-based models. Keep rate=0.5, window size=30.

Model	Train	Development	Test
LSTM^a^	54.47	41.43	42.32
Bidirectional LSTM	86.56	66.47	62.79
LSTM + Attention	68.69	52.71	54.21
Bidirectional LSTM + Attention	83.71	68.95	*65.72* ^b^

^a^LSTM: Long short-term memory.

^b^Best score on test data are highlighted in italics.

**Table 8:** Results (%) of the best-performing neural model (Bidirectional long short-term memory [LSTM] + Attention) on test set. Keep rate=0.5, window size=30.

Relation	Precision	Recall	F1-score
None	100	100	100
Dosage	78	80	79
Route	67	78	72
Frequency	61	76	68
Duration	54	69	61
Indication	32	32	32
Adverse	78	46	58
Severity	77	93	84
Overall	63.85	67.71	65.72

Table 7 shows the performance of variations of the neural models, including the attention-based and the bidirectional LSTM-based relation extractors. The attention-based models always performed better than their corresponding LSTM-based extractors. Furthermore, the bidirectional networks achieve much higher performance than the unidirectional ones. The bidirectional LSTM-based model yielded the highest F-1 training score. However, without the attention mechanism, this model appears to be overfitting. The best performance we obtained on the test set was a 65.72% overall F1-score for positive relation types, which was lower than the one we reported with SVM models. Table 8 shows the detailed test performance measures of the best-performing neural model (bidirectional LSTM + attention) for each relation type. Most of the relation types had F-1 scores above 70%, and *Severity* relation achieved the best performance of 84%. However, the scores for *Indication, Adverse,* and *Duration* relations were relatively low, with the *Indication* score being the lowest of 32%, which is consistent with SVM models. Nevertheless, the overall result is still promising, given the fact that no feature engineering was conducted and that the training set had only hundreds of examples.

For SVM models, we performed an efficient grid search over hyper-parameters, and this boosted performance substantially. However, we were not able to do the same for neural network models due to their computational complexity. Instead, we were able to perform a small random search for neural network parameters.

## Discussion

### Principal Findings

The bidirectional LSTM model with attention achieved the best performance among all the RNN variations, and additional features are shown to help boost the system performance. SVM model yields the best results, outperforming RNN models, but RNN models demonstrate great potential of significant improvement with more annotated data available.

Both the classic feature engineering-based SVM pipeline and the end-to-end neural network methods have advantages. The SVM model is able to exploit high-dimensional sparse representation (ie, *TF-IDF*), which has traditionally proven to be efficient in clinical NLP tasks. On the other hand, the neural model relies on dense low-dimensional representations that can possibly be constructed in unsupervised fashion from a large unlabeled text, eluding the complicated feature engineering efforts.

However, the neural models have a large number of training parameters that are tuned during training and are able to learn from a much larger dataset for better performance. For example, our bidirectional LSTM model has 1.4 million training parameters, so tuning this parameter set requires a large amount of data. Unfortunately, it is not trivial to obtain such labeled data in the clinical and biomedical domains. Our training data used in the experiments had hundreds of examples per relation type, which was a very small fraction compared with the bidirectional LSTM training parameters. In general, this is a disadvantage of deep learning approaches, and we empirically validated in our ADE relation identification tasks. In low-resource domains, such as the medical domain explored in this study, the focus of future work needs to be on data-efficient deep learning methods. In addition, the SVM relation extractor is easy to train and is robust with a small dataset. Training of the neural network-based relation extractor requires a graphic processing unit (GPU) and is computationally expensive. For example, 60 epochs of our attention model took 26 hours to complete on a GeForce GTX 980 GPU.

### Error Analysis

**Figure 3:**
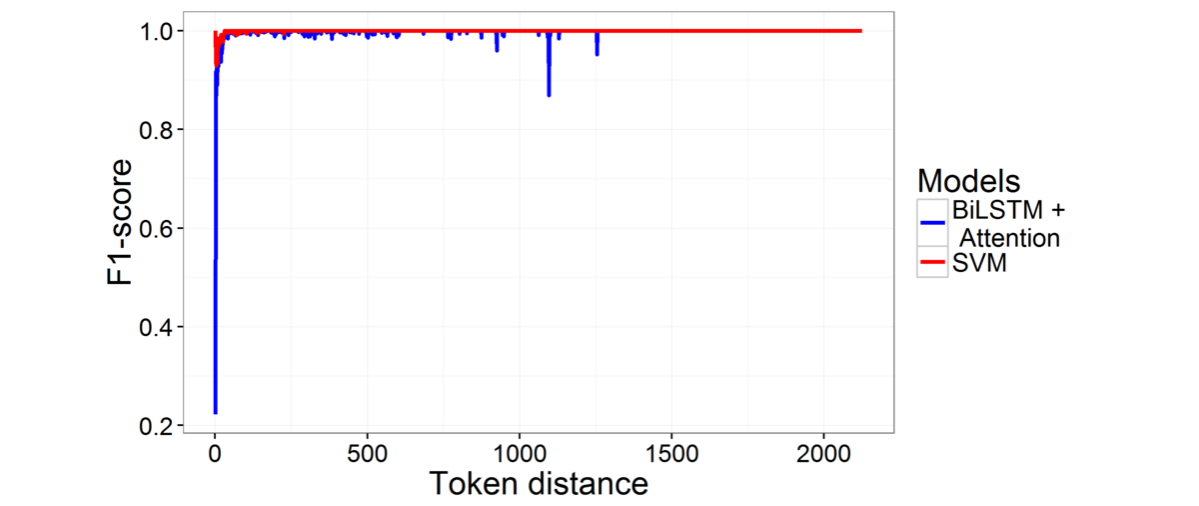
Test F1-score over relation distance. BiLSTM: bidirectional long short term memory; SVM: support vector machine.

We analyzed how well the SVM and attention models performed on short- and long-distance relations. Figure 3 plots the test F1-score of these models against relation distance. The bidirectional LSTM with attention did not perform well on short distance relations, and it was not stable. In contrast, SVM was very stable and performed well for those relations where the distances between the entities are long. Interestingly, the neural network performance decreased to 87% from 100% when the distance was 1100. The performance drop was due to false positives, and the generated negative examples were classified as positive by the model. However, these were the simple cases that even our rule induction classifier was able to easily detect. Therefore, we hypothesize that the neural network makes this obvious mistake because the context features, such as relation representations the model relies on, are not sufficient for the task. To justify this, we included a set of additional features in the neural network model. The token and mention distances and mention type features (in SVM models) were embedded and further used along with the dense-vector relation representations for classification.

By including these additional features in the neural model, we improved its best result from a 65.72% to a 77.35% F1-score. Table 9 provides a horizontal comparison of the different methods proposed in this paper. Inclusion of those features in the neural model yielded an approximately 12% improvement, and the performance gap between the neural model and SVM model was also reduced.

**Figure 4:**
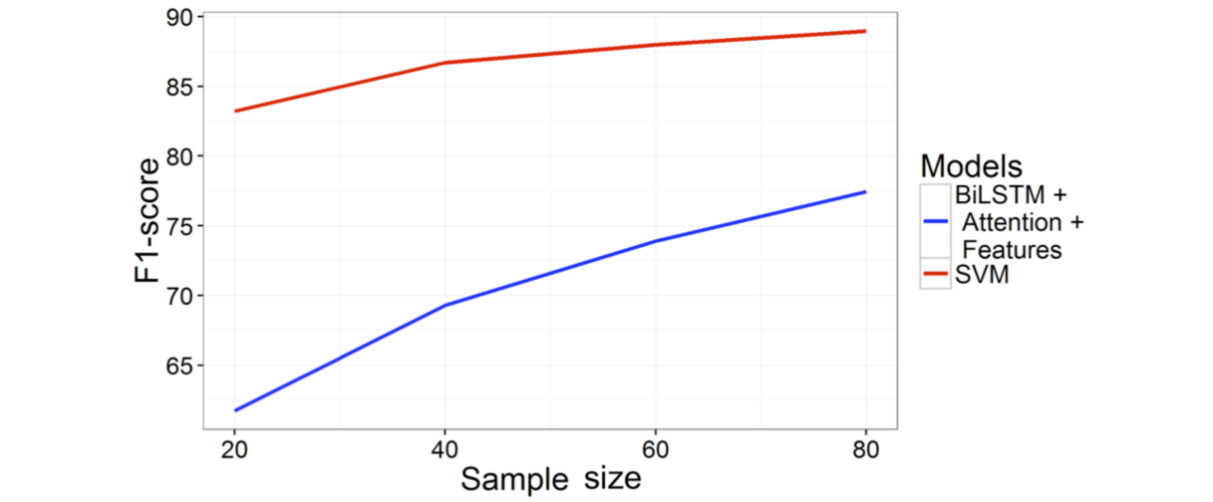
Test F1-score over varying training sample size. BiLSTM: bidirectional long short term memory; SVM: support vector machine.

We also conducted a set of experiments to show how the training data size affects the overall performance of the SVM and neural models. We created new training sets with stratified sampling rates of 20%, 40%, 60%, and 80% of the original training data. Both SVM and attention-based bidirectional LSTM models were trained on the new training sets and evaluated on the test data. In Figure 4, we display the test F1-scores of the models for different sample sizes. The SVM model achieved an F1-score greater than 80% even when trained on 20% of the data, but the performance of the neural model was only around 62%. This demonstrates that feature engineering approach may be preferred over deep learning models when less annotated data are available, as the hand-crafted features in the SVM model has encoded human knowledge, such as domain knowledge and various heuristics.

However, as the training dataset is increased, we can observe a firm improvement on the performances of the neural models. When we increased the training sample size from 20% to 80%, the neural model improved the test performance from ~62% to ~76, by almost 20%, whereas the improvement range for the SVM model was much smaller, around 8% F1-score. Therefore, the neural model has the potential to improve substantially if a larger training dataset is available.

### Limitations

One limitation of this study is that the size of the data in the experiment is relatively small, and more follow-up study is needed to further verify the findings on a larger dataset or other publicly available datasets (eg, i2b2 data although they only contain intrasentential relations) by exploring more RNN or CNN architectures, which we will investigate in our future work. In addition, the global attention in our LSTM model may not be sufficient to pinpoint important local context, especially for long-distance relations, and it is worth exploring more flexible attention mechanisms on this task.

**Table 9:** Comparison of different models in terms of overall F1-score.

Model	Train	Development	Test
Rule induction classifier	8.33	8.74	7.47
Bidirectional LSTM^b^	83.71	66.47	62.79
Bidirectional LSTM + Attention	86.56	68.95	65.72
Bidirectional LSTM + Attention + Features	88.14	77.77	77.35
SVM^a^ + Features	87.85	90.42	*89.1* ^c^

^a^LSTM: Long short-term memory

^b^SVM: support vector machines.

^c^Best score on test data are highlighted in italics.

### Conclusions

In this study, we created a new expert-annotated EHR corpus in the context of ADE relation identification, which will become a valuable resource and benchmark in drug safety surveillance research community. We, then, explored 3 different supervised machine learning models with different levels of complexity to identify 7 types of ADE-related clinical relations. Our results show that the SVM model with a rich feature set achieved the highest performance, surpassing both the rule induction model and the RNN models. The bidirectional LSTM model with attention achieved the best performance among the RNN models, and the additional features are shown to help boost the system performance. However, its performance remains substantially inferior to the performance of the SVM model, although RNN models demonstrate great potential of significant improvement with more annotated data available. Our results indicate that a rich feature set remains crucial for relation identification in clinical text, especially when the training size is small.

In the future, we will further explore different deep learning architectures (eg, multikernel CNNs, hierarchical RNNs, multilevel attentions) on this task for improved performance. Then, we plan to apply our system to EHRs on a large scale and derive meaningful insights to facilitate efficient and effective drug safety surveillance.

## Abbreviations

ADE: adverse drug event
CNNs: convolutional neural networks
EHR: electronic health record
FDA: Food And Drug Administration
FN: false negative
FP: false positives
GPU: graphic processing unit
HER: electronic health record
LSTM: long short-term memory
MLP: multilayered perceptron
NLP: natural language processing
RNN: recurrent neural network
SVM: support vector machines
TP: true positive
WVCs: Word Vector Classes
